# Canine breeds associated with gastric carcinoma, metaplasia and dysplasia diagnosed by histopathology of endoscopic biopsy samples

**DOI:** 10.1186/s13028-018-0392-6

**Published:** 2018-06-18

**Authors:** Marcus Vinicius Candido, Pernilla Syrjä, Susanne Kilpinen, Thomas Spillmann

**Affiliations:** 1 Department of Equine and Small Animal Medicine, Faculty of Veterinary Medicine, University of Helsinki, P.O. Box 57, 00014 Helsinki, Finland; 2 Department of Veterinary Biosciences, Faculty of Veterinary Medicine, University of Helsinki, P.O. Box 66, 00014 Helsinki, Finland

**Keywords:** Breed predisposition, Cancer, Dog, Dysplasia, Endoscopy, Gastric carcinoma, Gastroduodenoscopy, Metaplasia

## Abstract

**Background:**

Gastric carcinoma (GC) is a rather rare pathological finding in dogs, with the exception of some breeds which seem predisposed. The etiopathogenesis is largely unknown in dogs, whereas in humans GC often develops from gastric mucosal metaplasia and dysplasia. This study investigates whether dogs of certain breeds are more often subject to gastroduodenoscopy (GDS), and diagnosed with GC, mucosal metaplasia or dysplasia. A retrospective clinical database search was performed at the Veterinary Teaching Hospital at the University of Helsinki, Finland. The following inclusion criteria were applied to estimate relative risk for metaplasia/dysplasia and GC: dogs from pure breeds with at least five individuals subject to GDS with histopathology of gastric biopsies.

**Results:**

Between 2006 and 2016, from a total of 54945 canine patients presented, 423 dogs underwent GDS. Inclusion criteria were met in 180 dogs of 20 different pure breeds. Eight dogs had GCs (mean age = 9.8 ± 1.7 years): Belgian Tervuren (n = 4), Collie (n = 2), Golden Retriever (n = 1) and Jack Russel Terrier (n = 1). Fourteen dogs of eight breeds had gastric mucosal metaplasia or dysplasia. A log-binomial statistical model revealed that dogs in the following breeds had a significantly higher probability to undergo GDS than the others in the study population: Australian Terrier, Belgian Tervuren, Cairn Terrier, Collie and Siberian Husky. Belgian Tervuren was found at higher risk to be diagnosed with GC [RR = 19 (5.7–63.9; *P *< 0.0001)], as well as mucosal metaplasia/dysplasia [RR (7.6; 2.95–19.58; *P *< 0.0001)], as compared to the other breeds included. Shetland Sheepdog had an increased RR (5.83; 1.75–19.45; *P *= 0.0041) for metaplasia.

**Conclusions:**

The results indicate a very low incidence of GC in dogs. The Belgian Tervuren, however, appears as predisposed. The histopathologic descriptions of mucosal changes such as metaplasia and dysplasia were also rare, but were more frequent in the Belgian Tervuren. Previous reports of these changes in dogs are very scarce, but they might be presumably related to GC in dogs, as they are in humans. Future research should investigate the possible role of metaplasia and dysplasia in the development of GC in dogs, especially those of predisposed breeds.

## Background

Gastric carcinoma (GC) is a rare pathologic finding in dogs, as it corresponds to less than one percent of all neoplastic changes identified in dogs [1, 2]. The most common type of gastric neoplasm in dogs is adenocarcinoma, which is diagnosed at around 10 years of age, with initial clinical signs similar to those of other chronic gastrointestinal disorders [2–5]. Most gastrointestinal carcinomas in humans are known to evolve from superficial, flat changes classified as metaplastic or dysplastic according to their degree of cellular differentiation [6, 7]. GC is the fifth most common type of cancer in people worldwide, and endoscopic biopsy is essential for diagnostic and staging [8]. Visual enhancement techniques such as chromoendoscopy and narrow band imaging are often applied for cancer screening and surveillance, as they can help improve the diagnostic yield of discrete mucosal changes [9]. The pathogenesis in humans is complex and not fully understood, but in approximately 90% of the cases it involves a multistep, multifactorial process. The progression from normal mucosa to chronic gastritis, further evolving to atrophic gastritis, intestinal metaplasia, dysplasia and adenocarcinoma, occurs over several years [10]. However, challenging this classic histogenetic pathway in which metaplasia and dysplasia are well-recognized pre-neoplastic changes, human GC can also develop from otherwise normal gastric mucosa. Diffuse gastric cancer, for example, is rare and often develops in a hereditary setting from foci of superficial signet ring carcinoma in situ [11]. Gastric infection with *Helicobacter pylori* plays a major role among environmental factors contributing to an increased risk of gastric cancer in humans [12], along with dietary factors including high intake of salt and preserved or smoked food (especially meat products), and low consumption of fresh fruit and vegetables [13].

The etiology of GC in dogs is still unknown, and it is suspected to be as complex as in humans [14]. The cardinal histological changes in canine gastric carcinogenesis remain undetermined, and the correlation between gastric polyps, *Helicobacter* spp. and GC has not yet been demonstrated [5, 15, 16]. A study on 14 Norwegian Lundehund dogs describes findings of mucous metaplasia in association with varying degrees of atrophic gastritis and intestinal lymphangiectasia. Additionally, four of those dogs had gastric neoplasia, which suggests that the changes represent a single pathological process [17]. In a further study with a total of eight Lundehunds with gastric neoplasia, it seems that such tumors are rather of neuroendocrine origin (derived from enterochromaffin-like-cells), possibly secondary to gastric atrophy and hypergastrinemia [18]. However, canine GC is not always associated with atrophic gastritis and comprehensive studies demonstrating the progression of atrophy towards metaplasia, dysplasia and gastric cancer in dogs are still lacking.

Previous retrospective studies based on data from canine cancer registry, records from specialists, pathology laboratories and breeders have shown breed predisposition to GC in the Belgian Tervuren, Bouvier des Flandres, Groenendael, Collie, Chow–Chow, Poodle, Norwegian Elkhound and a few other breeds, which implies a genetic background for the disease [1, 3, 4, 19, 20]. In Finland, information about breed association to gastric cancer has been missing.

The present study was conducted to investigate which pure breeds are most commonly subject to gastroduodenoscopy (GDS) at the Veterinary Teaching Hospital of the University of Helsinki, a national referral center. The most important objective was to determine the probability of dogs in different breeds undergoing endoscopy with mucosal biopsy for histology to be diagnosed with gastric carcinoma, metaplasia or dysplasia, as a possible evidence of breed predisposition also in Finland. The overall aim of this study was to gather information about gastric cancer and metaplastic or dysplastic changes as recognizable features in canine patients undergoing endoscopy with gastric mucosal biopsy for histology. This study relies on the hypothesis that, in the clinical setting of an endoscopic unit, certain breeds can be found affected with GC and mucosal metaplasia/dysplasia more commonly than others. The study therefore aims at investigating a possible association between individual breed, submission to GDS, and diagnosis of gastric carcinoma and metaplastic or dysplastic mucosal changes in histopathology.

## Methods

The study was designed as a retrospective cohort on the diagnosis of GC, metaplasia and dysplasia in dogs subjected to GDS. The source population of this study comprises all dogs presented at the Veterinary Teaching Hospital between January 2006 and December 2016. A computerized database search was performed on the records throughout this period. The study population included dogs which belong to a pure breed having a minimum of five dogs subject to GDS with complete histopathological report based on gastric biopsies. The Belgian Tervuren is herein regarded as a breed on its own. The breed Collie includes both rough and smooth haircoat varieties (Border Collie and Shetland Shepherd being separate pure breeds).

The endoscopic biopsy samples were collected in a standardized fashion: at least eight gastric mucosal fragments were taken onto wood chips and then immediately immersed (upside down) in 10% neutral buffered formalin. After 24 h fixation, the biopsies were processed, paraffine-embedded, sectioned at 4 microns and stained with hematoxylin and eosin (HE). Special staining such as periodic acid-Schiff (PAS) or Warthin–Starry (WS) were applied when indicated.

The endoscopic mucosal biopsy samples were examined at the University of Helsinki by veterinary pathologists adhering to a standardized assessment protocol since 2006. The standard reporting template included the following parameters as main criteria for histopathological evaluation of the stomach: number of biopsy samples examined, epithelial surface characterization, amount of intraepithelial lymphocytes, presence of *Helicobacter*-like organisms, characterization and amount of mononuclear and granulocyte infiltration in lamina propria, presence of lymphoid follicles, characterization of fundic glands, and whether pepsinogen and HCl-producing cells are identified regularly, or else substituted by mucous cells (metaplasia) [21]. Gland structure was also evaluated regarding presence of fibrosis, glandular nesting and structural changes which could be related to dysplasia. The World Small Animal Veterinary Association standards and guidelines were applied from 2008 onwards and the template was updated accordingly, regarding grading of inflammatory changes (0–3: normal, mild, moderate, severe) [22, 23]. The diagnoses were given under the supervision of, and in consensus with, a board-certified pathologist (PS).

The complete histopathological record of endoscopic biopsies from each patient of the study population was revisited for findings consistent with either gastric carcinoma (GC), dysplasia or metaplasia, in addition to other features [21–23]. Histologic sections from the patients presenting these specific lesions were re-examined by a board-certified pathologist (PS) for verification of the diagnosis and consistency of the results.

### Statistical methods

All of the outcomes tested were binary, because the presence or absence of GDS, GC or metaplasia/dysplasia was considered for each case studied. A log-binomial model was applied for each outcome variable, and the probability of presence was modelled with 95% confidence intervals (CI). The models were re-run separately for each breed in the analysis population comparing the breed of interest to other breeds. Relative probability to undergo GDS (numerically equivalent to relative risk) was calculated for each selected breed in comparison to all other canine patients visiting the hospital in the period (source population). The individual histopathological diagnosis from dogs of breeds meeting the inclusion criteria (study population) was considered for the calculation of relative risk (RR) to be diagnosed with gastric metaplasia/dysplasia, or carcinoma, and computed for each breed in comparison to the other breeds included. *P*-values < 0.05 were considered significant. All analyses were done using SAS^®^ System for Windows, version 9.3 (SAS Institute Inc., Cary, NC, USA).

## Results

### Pure breeds undergoing gastroduodenoscopy (GDS)

Between 2006 and 2016, the source population included a grand total of 54,945 canine patients being presented at the hospital. The study population was recruited out of 423 dogs which underwent GDS for diagnostic reasons, 30 of which being mixed-breed dogs. The inclusion criteria mentioned above were met by 180 dogs representing 20 different pure breeds. The study population consisted of 75 intact males, 26 castrated males, 46 intact females and 33 sterilized females, which underwent GDS at the mean age of 5.3 ± 3.4 years (n = 180). These dogs correspond to 43% of all GDS patients in the period (Table 1). Twelve other individual dogs of the breeds included had already been excluded from the study either because histopathological information was lost (n = 3) or no biopsies were taken during GDS (n = 9). Six of those nine dogs had gastric foreign bodies removed (plastic; wood; needles; stone) and one presented biliary tract changes and underwent endoscopic retrograde cholangiopancreatography followed by surgical removal of the gallbladder.

**Table 1:** Study population: breeds with at least five dogs undergoing gastroduodenoscopy (GDS) and histopathological examination of biopsy samples, selected for inclusion from the source population of all dogs presented between 01.01.2006 and 31.12.2016

Breed	Total dogs presented	Dogs undergoing GDS
Australian Terrier	245	5
Belgian Tervuren	196	9
Border Collie	430	5
Cairn Terrier	522	11
Chihuahua	1729	5
Collie	673	27
Dachshund	1609	7
French bulldog	845	7
German Shepherd	2110	18
Golden Retriever	1216	15
Hovawart	328	5
Jack Russell Terrier	1149	7
Labrador retriever	2124	14
Miniature Schnauzer	1003	7
Parson Russell Terrier	496	6
Rottweiler	794	10
Shetland Sheepdog	734	5
Siberian Husky	219	5
Staffordshire Bull Terrier	592	6
West Highland White Terrier	690	6
Dogs in the breeds above	17,704	***180***
Other breeds/mixed (not included)	37,241	243^a^
All dogs in the period	*54,945*	423^b^

Study population is indicated in bolditalic

Source population is indicated in italic

^a^Includes individual dogs from the selected breeds which were excluded from the study due to lack of histopathological diagnosis (foreign body retrieval, no biopsy taken or biopsy lost to follow-up)

^b^Total number of GDS patients in the period

**Fig. 1:**
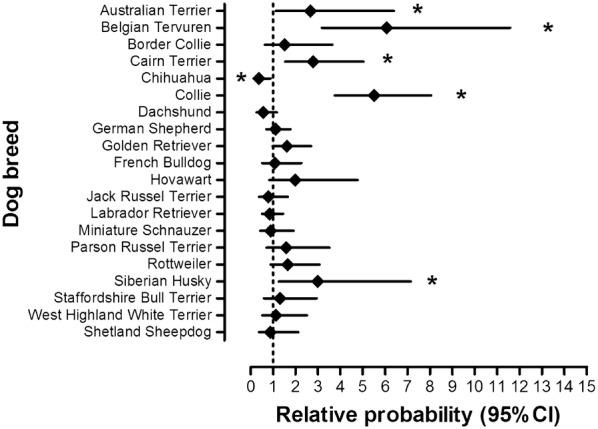
Relative probability to undergo GDS for dogs of the selected breeds in comparison to all other dogs in the source population. *The difference is statistically significant (*P *< 0.05)

When comparing the study population with the source population, a log-binomial model revealed significantly higher relative probability to undergo GDS in the following breeds as compared to dogs of other breeds (RR; 95% CI lower–higher; *P* value): Australian Terrier (2.67; 1.12–6.39; *P *= 0.0274), Belgian Tervuren (6.07; 3.18–11.58; *P *< 0.0001), Cairn Terrier (2.78; 1.54–5.03; *P *= 0.0007), Collie (5.5; 3.75–8.06; *P *< 0.0001) and Siberian Husky (2.99; 1.25–7.15; *P *= 0.0138). Chihuahuas underwent GDS significantly less often than dogs of other breeds (0.37; 0.15–0.89; *P *= 0.0261) (Fig. 1).

### Gastric carcinoma

**Fig. 2:**
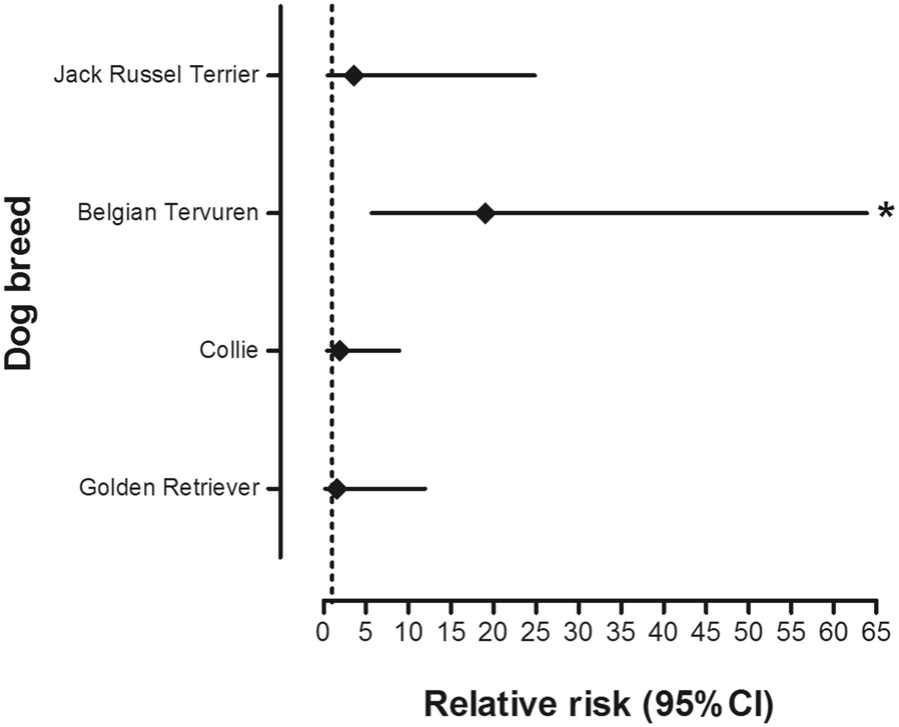
Relative risk to be diagnosed with gastric carcinoma for selected breeds in comparison to the other breeds in the study population. *The difference is statistically significant (*P *< 0.0001)

Eight of the dogs included in the study were diagnosed with gastric adenocarcinomas, which was the only type of gastric neoplasm seen in the study population. Affected dogs belonged to the following breeds: Belgian Tervuren (n = 4, including one signet-cell carcinoma), Collie (n = 2), Golden Retriever and Jack Russel Terrier (Table 2). Five of them were female and three were male. Concerning age at diagnosis of GC, the youngest patient was 7.8 years old when the examination was performed (mean age = 9.8 ± 1.7 years; see also Table 3). The Belgian Tervuren was found at much greater relative risk for being diagnosed with GC from histopathological examination of endoscopically obtained gastric biopsies (33.4; 6.43–173.36; *P *< 0.0001) in comparison to other breeds (Fig. 2).

**Table 2:** Number of dogs in each breed undergoing gastroduodenoscopy (GDS), and diagnosed with gastric carcinoma (GC), dysplasia or metaplasia from endoscopic biopsy samples taken from 01.01.2006 to 31.12.2016

Breed	GDS	GC	Dysplasia or metaplasia
Belgian Tervuren	9	4	4
Collie	27	2	2
Dachshund	7	–	1
Golden Retriever	15	1	2
Hovawart	5	–	1
Jack Russell Terrier	7	1	–
Shetland Sheepdog	5	–	2
Siberian Husky	5	–	1
Staffordshire Bull Terrier	6	–	1
Total in these breeds	86	8	14

**Table 3:** Breed, age (years), sex and concomitant changes found in each of the dogs diagnosed with gastric carcinoma (GC)

Breed	Age	Sex	Inflammatory change	Structural change
Belgian Tervuren (n = 4)	7.8	F	Moderate chronic LP G	Glandular dysplasia
	9.7	F	Moderate chronic LP G	–
	9.2	M	Moderate chronic LP G with neutrophilic component and severe gland hyperplasia	Glandular dysplasia, epithelial dysplasia and atypia
	11.0	M	Severe chronic LP G	–
Collie (n = 2)	8.6	F	Moderate chronic LP G	–
	8.5	Fs	Moderate chronic atrophic G	Mucinous metaplasia
Golden Retriever	10.8	F	Severe chronic LP G with neutrophilic component and ulcerative mycotic G	–
Jack Russell Terrier	12.9	Mc	Severe gland hyperplasia, mild LP G	–

*LP* lymphoplasmacytic, *G* gastritis, *Fs* sterilized female, *Mc* castrated male

### Metaplasia and dysplasia in gastric mucosa

**Fig. 3:**
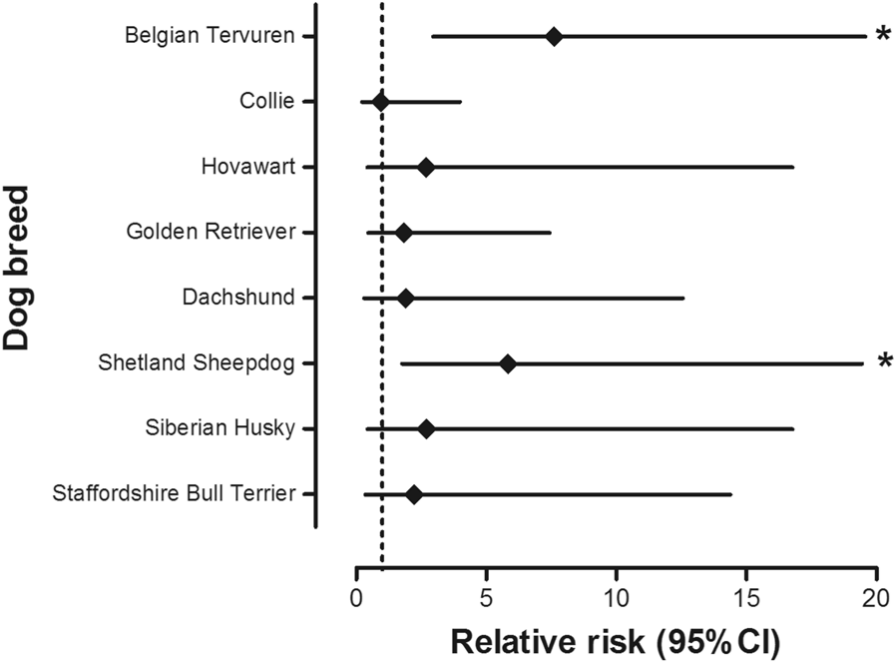
Relative risk to be diagnosed with gastric mucosal metaplasia or dysplasia for selected breeds in comparison to the other breeds in the study population. *The difference is statistically significant (*P *< 0.005)

Changes histologically described as metaplastic or dysplastic were seen in 14 dogs in the following breeds: Belgian Tervuren (n = 4), Collie (n = 2), Golden Retriever (n = 2), Shetland Sheepdog (n = 2), Dachshund (n = 1), Hovawart (n = 1), Siberian Husky (n = 1) and Staffordshire Bull Terrier (n = 1). The mean age when these patients underwent GDS was 7.9 ± 2.6 years. Significant statistical differences were found for the diagnosis of either metaplasia or dysplasia in Belgian Tervuren compared to other breeds: RR (7.6; 2.95–19.58; *P *< 0.0001). The Shetland Sheepdog was found at significantly increased RR (5.83; 1.75–19.45; *P *= 0.0041) for metaplasia (dysplasia was not seen in this breed) compared to dogs of other breeds (Fig. 3).

### Endoscopic and histopathological diagnosis

**Fig. 4:**
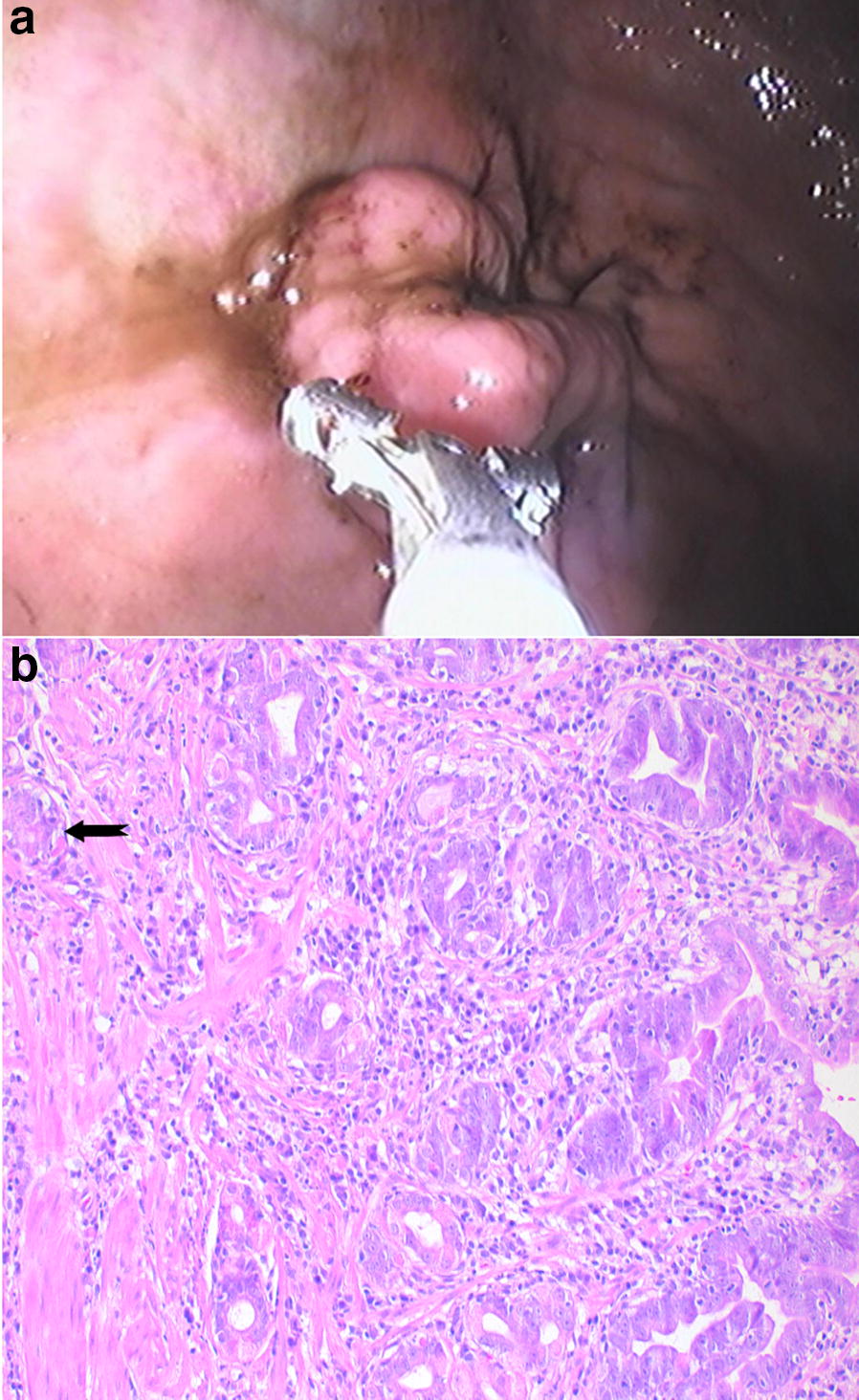
Endoscopic and corresponding histologic images of an 8-year-old female Belgian Tervuren showing gastric adenocarcinoma. **a** White light endoscopic image of a gastric adenocarcinoma. Histology: **b** Well differentiated gastric adenocarcinoma with invasion of the *muscularis mucosae* (arrow). HE stain, Obj. 20×

**Fig. 5:**
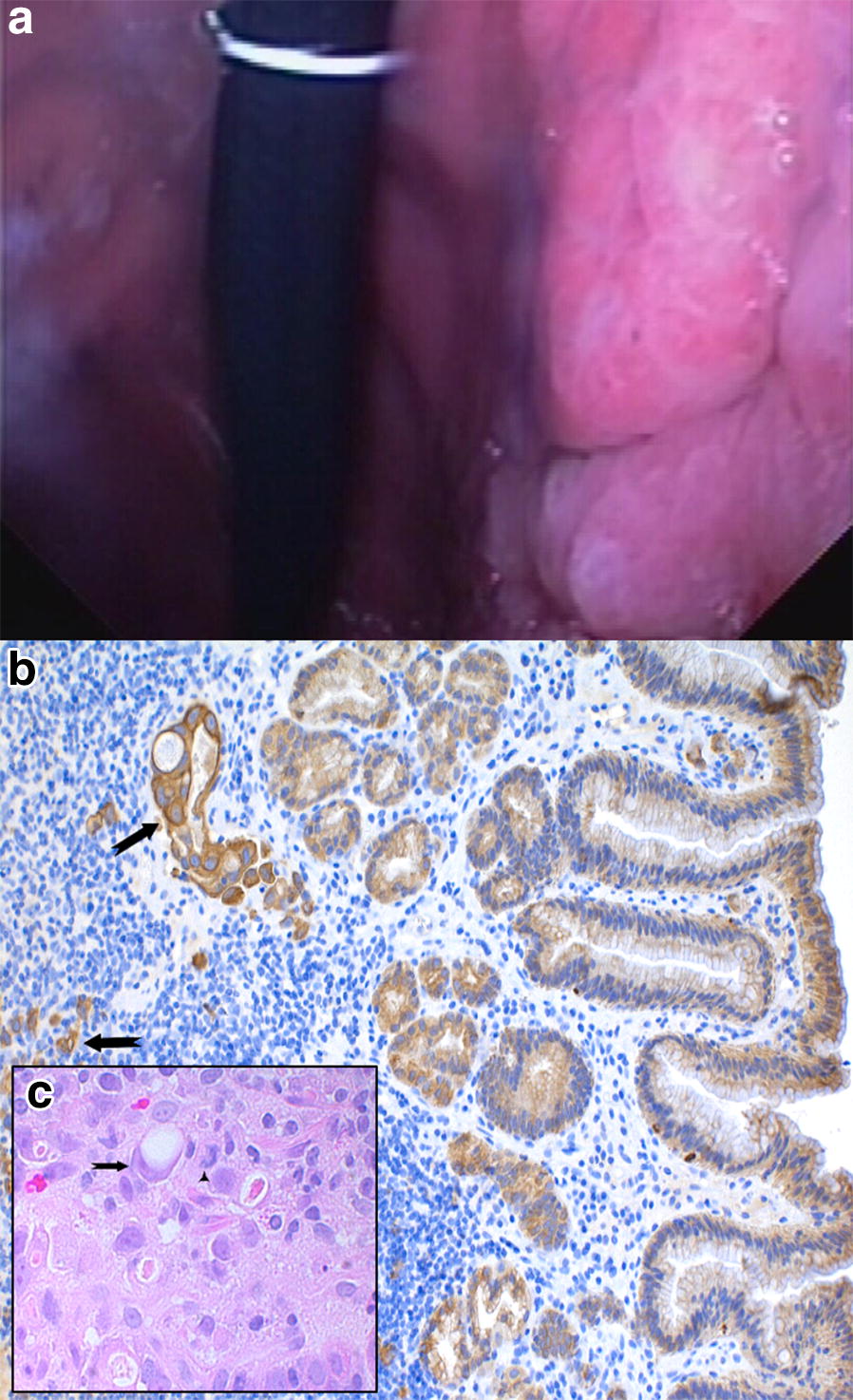
Endoscopic and corresponding histologic images of a 10-year old female Belgian Tervuren representing gastric adenocarcinoma. **a** White light endoscopic image of a diffuse mass in the stomach. Histology: **b** Invasive gastric adenocarcinoma, with atypical tubular structures (arrow) and single cell invasion of deeper layers of the gastric wall (arrow), and **c** classical signet ring cells (arrow). **b** IHC cytokeratin stain, Obj. 20×. **c** HE stain, Obj. 40×

**Fig. 6:**
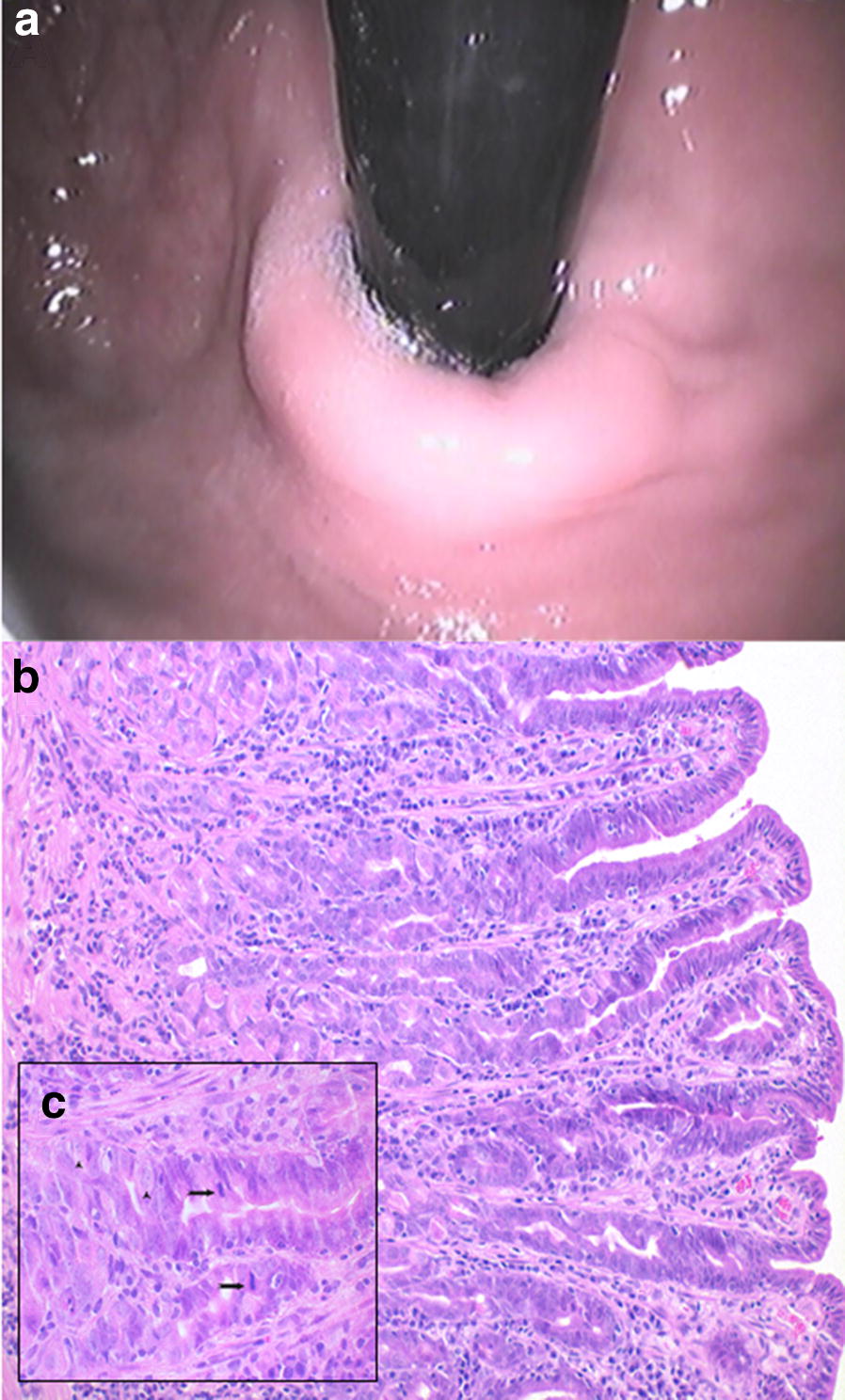
Endoscopic and corresponding histologic images of a 9-year-old castrated male Dachshund representing metaplasia. **a** White light endoscopic image of gastric fundus with discrete mucosal changes. Histology: **b** Mucous/intestinal metaplasia within the gastric fundus; elongated hyperplastic mucous neck regions of fundic glands and diffuse moderate lymphoplasmacytic gastritis. **c** Increased mitotic figures within the hyperplastic mucous cells of the glandular neck area (arrow). Scattered parietal cells are visible (arrowhead) within the basal area of the glands. HE stain, **b** Obj. 20×, **c** Obj. 40×

**Fig. 7:**
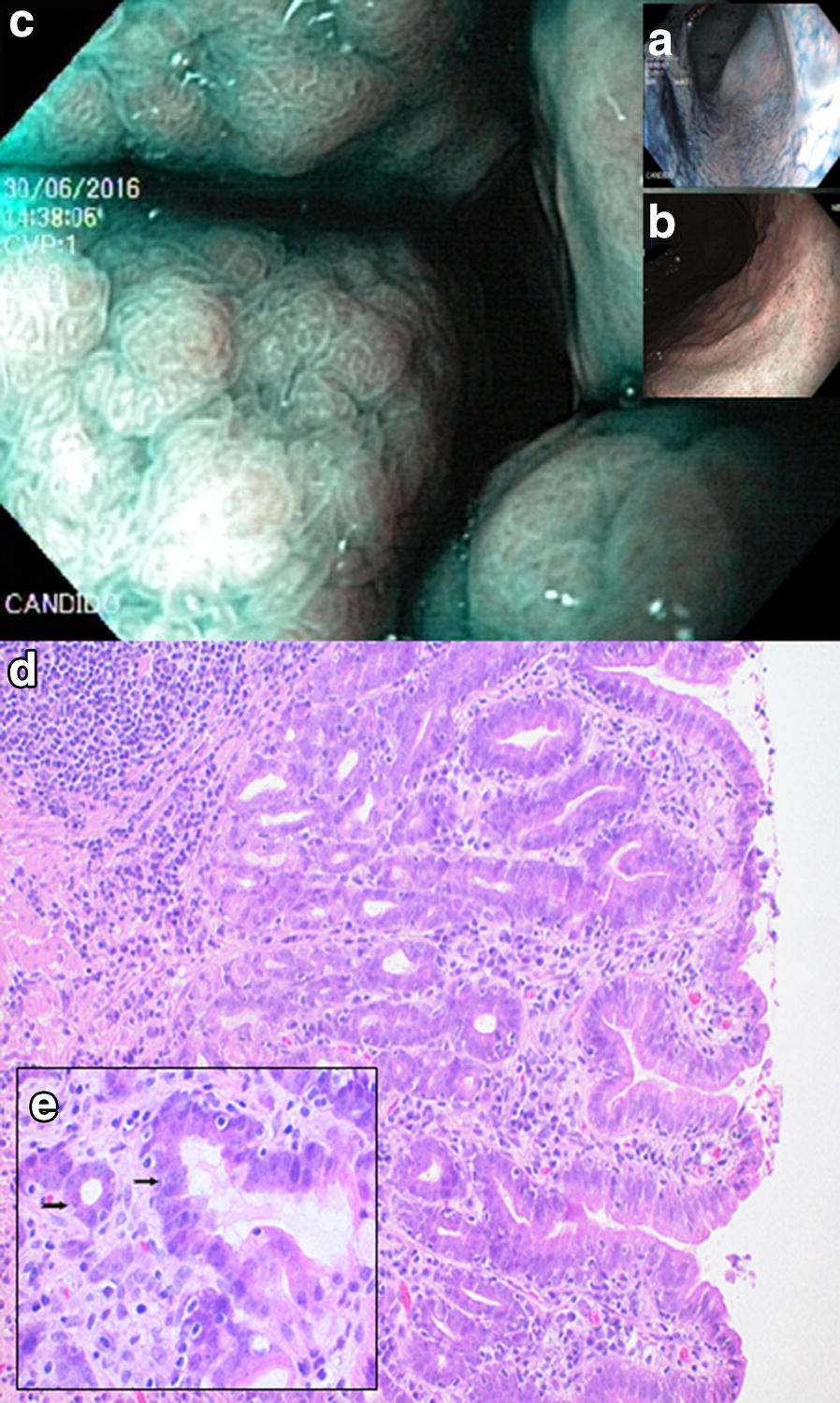
Endoscopic and corresponding histologic images of a 9-year-old female Belgian Tervuren showing dysplasia. Endoscopic images (**a**, **b**, **c**) show diffuse mucosal texture irregularity along gastric fundus and body: **a** chromoendoscopy (CE), **b** narrow band imaging (NBI), **c** CE and NBI combined. Histology: **d** dysplasia of the fundic glands, with glandular dilatation and distortion. **e** Cellular dysplasia with flattening of the glandular cells and loss of cellular polarity (arrows). HE stain, **d** Obj. 20×, **e** Obj. 40×

The final diagnosis considered for all of the analyses was achieved by endoscopic examination and sampling, followed by histopathological confirmation. All dogs presenting the changes of concern were also found with concomitant inflammatory changes (Table 3). Each set of sample images shown in this article correspond to a single patient, respectively diagnosed as carcinoma (Figs. 4, 5), metaplasia (Fig. 6) or dysplasia (Fig. 7). Chromoendoscopy and narrow band imaging were applied in one Belgian Tervuren presented with chronic vomiting, whose brother from the same litter had been euthanized due to a massive GC. The most prominent endoscopic and respective histopathological changes found in this patient are in Fig. 7.

## Discussion

Some dog breeds (namely Australian Terrier, Belgian Tervuren, Cairn Terrier, Collie and Siberian Husky) underwent GDS significantly more often as compared to the other breeds in this study. Chihuahua was scoped significantly less often than others; this breed with a relatively small body size is rather common in the source population. These findings remain largely unexplained, however breed distribution (Table 1) and relatively low overall frequency of GDS (low sample sizes) may have influenced the results. It is not clear whether it was the severity of gastrointestinal signs alone, or any other issues that might have influenced the probability for certain breeds to undergo GDS. It would be reasonable to speculate that an expected breed predisposition to GC, as previously reported for the Belgian Tervuren [1, 3, 19], might potentially bias the approach of clinicians and owners towards submitting patients of this breed to endoscopy earlier, or more often. The fact that dogs of certain breeds undergo GDS more often might as well indicate a higher prevalence of gastrointestinal disorders in general, which may warrant more extensive studies in such breeds.

The Belgian Tervurens that underwent GDS in this study were consistently found at much higher risk of being diagnosed with GC than dogs of any other breed. This finding is in accordance with previous studies that used pathological records and cancer registry data of other countries such as Italy, Netherlands or Norway, in which the Belgian Tervuren were also considered to be highly predisposed to GC [1, 3, 19].

Gastric metaplasia or dysplasia were almost as rarely seen as GC, with significantly higher relative risks found for the Belgian Tervuren and Shetland Sheepdog. Although statistical significance could not be confirmed for every breed, both types of changes were found in Belgian Tervuren, Collie and Golden Retriever, sometimes in the same patient. Nonetheless, metaplasia and dysplasia can present as discrete, flat changes that are easily overlooked and possibly underdiagnosed [24], especially when considering the limitations of current white light endoscopy modalities available and non-directed sampling procedures in veterinary medicine. Visual enhancement techniques such as chromoendoscopy and narrow band imaging (Fig. 7) may help improve the diagnostic yield of discrete mucosal changes [9].

One of the major challenges in veterinary gastroenterology is that the extension and severity of macroscopic changes seen during endoscopy are not always consistent with the respective histopathological findings from endoscopic biopsy [25]. Cognitive aspects including specific knowledge, endoscopist experience and training, standardized grading systems, image quality and meticulous examination techniques are fundamental for the detection of subtle lesions [26]. In this study, the biopsies and histologic sections were prepared and then examined according to a uniform in-house standard. Such protocols help to control the risk of inaccuracy related to lack of uniformity on sample quality and to interobserver variation in histopathological evaluation. Such issues have been reported as potentially detrimental when histologic material or reports originated from several different institutions are utilized [27, 28].

The diagnosis of metaplastic or dysplastic changes in breeds with predispositions to GC might indicate a stepwise progression in dogs, comparable to the better known pathway towards GC in humans [10]. Studies in Lundehund dogs with GC ponder on a similar pathogenesis [17, 18]. This might be the case especially for the Belgian Tervuren with its significantly higher relative risk for the suspected pre-neoplastic changes, along with its marked predisposition for GC. This hypothesis might be further explored by staging suspected lesions and screening dogs selected from predisposed breeds. Research should aim at applying more advanced endoscopic approaches such as chromoendoscopy or narrow band imaging, which are proven modalities in humans for improving the targeted sampling of lesions [29] that might be easily overlooked when using white light endoscopy alone. Metaplasia and dysplasia have not been consistently studied in canine gastroenterology and their potentially relevant role in the pathogenesis of canine GC must be further investigated, whereas in humans well-established staging protocols and advanced therapeutic approaches are routinely applied [30]. While standard guidelines for histopathology evaluation remain fluid in veterinary medicine, novel immune staining techniques and genetic examination of biopsy samples may provide valuable opportunities for both research and diagnosis in the near future [31].

The retrospective nature of the study and the sole inclusion of dogs that had undergone GDS led to some limitations that possibly influenced the interpretation of our results. This study did not include GC diagnosed after necropsy or full-thickness biopsy but rather focused on the findings of endoscopic biopsy samples. This approach resulted in a small case load, as endoscopy is typically performed in a relatively small proportion of dogs in the clinical population, thus it relies on specific indications and also the owner’s compliance. A possible bias of indication for endoscopic procedures by different clinicians (e.g. awareness of certain breed predispositions), over such an extended time period, cannot be ruled out.

It is also worth mentioning that even in this selected population of canine patients with clinical signs of gastrointestinal disease, GCs were seen only in few individuals (8/180 = 4.4%), which further corroborates the rarity of the condition. The frequency of disease in this study cannot be directly compared with previous studies that used different approaches. However, the low frequency of diagnosis and the advanced age at diagnosis of GC in dogs found in this setting are in agreement with previous reports [1–4, 14].

Gaining knowledge about the actual prevalence of metaplasia and dysplasia in dogs may help to determine their possible role as pre-neoplastic changes. Furthermore, patient follow-up after the diagnosis of metaplastic and dysplastic changes might contribute to the knowledge about canine gastric carcinogenesis. Multi-center, prospective studies that utilize standardized approaches could help overcome the limitations related to reduced sample sizes in veterinary research, especially with regard to uncommon disease conditions like the ones reported in this study.

## Conclusions

Belgian Tervurens, Collies, Siberian Huskies, Cairn Terriers and Australian Terriers underwent GDS more often than dogs of other breeds in our study population. The Belgian Tervurens were found to be at much higher risk (19-fold) of being diagnosed with GC than dogs of other breeds, which further corroborates predisposition for GC in this breed. Metaplastic or dysplastic changes were slightly more frequent than GC. Although no causal relationship could be established thus far, the presence of lesions of all three types in breeds such as the Collie, the Golden Retriever, and chiefly the Belgian Tervuren, even in the face of these low case numbers, might be a sign of associated pathogenesis requiring further investigation.
