# Correction to: Canine breeds associated with gastric carcinoma, metaplasia and dysplasia diagnosed by histopathology of endoscopic biopsy samples

**DOI:** 10.1186/s13028-020-00520-8

**Published:** 2020-05-29

**Authors:** Marcus Vinicius Candido, Pernilla Syrjä, Susanne Kilpinen, Thomas Spillmann

**Affiliations:** 1 Department of Equine and Small Animal Medicine, Faculty of Veterinary Medicine, University of Helsinki, P.O. Box 57, 00014 Helsinki, Finland; 2 Department of Veterinary Biosciences, Faculty of Veterinary Medicine, University of Helsinki, P.O. Box 66, 00014 Helsinki, Finland

## Correction to: Acta Vet Scand (2018) 60:37 10.1186/s13028-018-0392-6

Following publication of the original article [1], the authors identified an error in the numbers reported in the Gastric Carcinoma section. The correct numbers are marked in bold below:

“The Belgian Tervuren was found at much greater relative risk for being diagnosed with GC from histopathological examination of endoscopically obtained gastric biopsies (**19; 5.7-63.9**; *P* < 0.0001) in comparison to other breeds (Fig. 2).”
